# Aqueous Neem Extract Versus Neem Powder on *Culex quinquefasciatus*: Implications for Control in Anthropogenic Habitats

**DOI:** 10.1673/031.011.14201

**Published:** 2011-10-31

**Authors:** Andreas A. Kudom, Ben A. Mensah, Mary A. Botchey

**Affiliations:** Department of Entomology and Wildlife, University of Cape Coast, Cape Coast, Ghana

**Keywords:** *Azadirachta indica*, biopesticide, local preparation, mosquito control, urban areas

## Abstract

Control programs using conventional insecticides to target anthropogenic mosquito habitats are very expensive because these habitats are widespread, particularly in cities of most African countries. Additionally, there are serious environmental concerns regarding large-scale application of most conventional insecticides. Clearly there is a need for alternative methods that are more effective, less expensive, and environmentally friendly. One such method would be the application of preparations made from parts of the neem tree, *Azadirachta indica* A. Jussieu (Sapindales: Meliaceae). In this study, aqueous crude extracts and crude powder were prepared from different parts of neem, and the efficacies of the preparations on juvenile stages of *Culex quinquefasciatus* Say (Diptera: Culicidae) were evaluated in the laboratory. When larvae were exposed to a concentration of 0.1 g/mL extract for 24 hours, percent mean mortality (± SE) was 72.7 plusmn; 1.8 for the bark, 68.7 ± 1.6 for fruits and 60 ± 1.6 for leaves. These means were not significantly different (χ^2^ = 4.12; df = 2; *p* = 0.127). At a concentration of 0.01 g/mL, > 95% of the larvae died within 24 hours of exposure to powdered neem leaf, but it took 120 hours to reach the same level of larval mortality in aqueous leaf extract. The crude extract slowly inhibited the growth and development of mosquitoes while the crude powder acted more as a barrier; the mosquitoes probably died from suffocation. However, both types of preparations can be made and used by local people to control mosquito breeding in anthropogenic habitats, especially in urbanized areas.

## Introduction

*Culex quinquefasciatus* Say (Diptera: Culicidae) is an important vector of filariasis and many other diseases (Service 2008), and can cause considerable irritation with their bites. Also, the high level of pyrethroid-resistance in *Cx. quinquefasciatus* in Africa (Chandre et al. 1998; Corbel et al. 2007) represents an obstacle to malaria prevention, as people may not appreciate the personal protective effect of insecticide treated nets if these nets do not kill *Culex* mosquitoes on contact.

*Cx. quinquefasciatus* is best controlled by improved sanitation and the installation of modern sewage systems (Service 2008); however, this is often not feasible, and insecticidal measures must be employed. In most areas, *Cx. quinquefasciatus* is resistant to a wide range of insecticides (Chandre et al. 1998; Corbel et al. 2007), limiting the choice of chemicals that can be used. These concerns have contributed to the search for eco-friendly larvicides; in recent years, the most prominent phytochemical pesticides have been those based on neem products (Mulla and Su 1999).

The neem tree, *Azadirachta indica* A. Jussieu (Sapindales: Meliaceae), has well-known insecticidal (Wandscheer et al. 2004) and insect growth regulatory (IGR) constituents (Sukumar et al. 1991; Batra et al. 1998; Copping and Menn 2000), and has been used for centuries in India (Schmutterer 1990). Various neem products have been studied extensively for their phytochemistry and exploitation in pest control programs (Mulla and Su 1999). A number of bioactive components have been isolated from various parts of the neem tree, azadirachtin being the major component. Several other active ingredients have been isolated from different parts of the neem tree including salannin, meliantriol, and nimbin (Mulla and Su 1999). Two new triterpenoids (22, 23-dihydronimocinol and des-furano-6-alpha-hydroxyazadiradione) were isolated from a methanolic extract of the fresh leaves of *A. indica* along with a known meliacin, 7-alpha-senecioyl-(7-deacetyl)-23-O-methylnimocinolide (Siddiqui et al. 2003).

Numerous studies investigating mosquitocidal properties of the neem leaves, fruits, and bark have been carried out (Awad and Shimaila 2003, Wandscheer et al. 2004; Howard et al. 2009; Martínez-Tomás et al. 2009). However, most neem extracts require expertise to prepare, making it difficult for local people to adopt and use neem-based insecticides on their own. In this study, the efficacies of different locally prepared neem extracts, i.e., not commercial preparations, on juvenile stages of *Cx. quinquefasciatus* were assessed in the laboratory.

Similar to other areas in Africa, anthropogenic activities in Ghana can create suitable mosquito breeding habitat. *Cx*. *quinquefasciatus* often breeds in water held in improper drainage systems or choked gutters. The use of conventional insecticides to control the breeding of mosquitoes in anthropogenic habitats would prove expensive, especially in cities where such habitats are numerous. Besides the financial cost, there are environmental costs of large-scale application of conventional insecticides. The aim of this study was to assess the effectiveness of preparations that are relatively inexpensive, easy to prepare locally, and which could be applied to these habitats to control the breeding of mosquitoes.

## Materials and Methods

### Larval collection

Field strains of *Cx. quinquefasciatus* larvae were collected from stagnant water at Apewosika (05° 06.532′ N, 01° 17.407′ W), a community near the University of Cape Coast, Ghana. In the laboratory, larvae were separated into 1^st^, 2^nd^, 3^rd^ and 4^th^ instars. Both the larvae and pupae were fed on powdered cat food (Whiskas, www.whiskas.com) and were maintained at 70–85% RH and temperature of 27±2 °C.

### Extract preparation

Leaves, fruits, and bark were collected from neem trees on the campus of the University of Cape Coast and used to prepare a water extract: neem parts were weighed and emulsified in 1000 mL of distilled water with a blender (Philips, www.philips.com), and the suspension was sieved through a muslin cloth. A powder was prepared by drying whole fruits or leaves at 35–40 °C in an oven and grinding them (Philips) to form a powder.

### Comparative efficacy of fruit, bark, and leaf extract

Bioassays were carried out on the mosquitoes to compare the effectiveness of extracts of the bark, fruit, and leaf of neem. High concentrations of extracts were used against 3^rd^ and 4^th^ instar larvae in the bioassays. Ten late instar larvae were placed in a 150 mL plastic cup with 100 mL distilled water and 40 mL of neem extract. Three concentrations were prepared for each plant part and each concentration was replicated 15 times (Table 1). Distilled water was used as a control.

### Effect of leaf extract on different instars

Further studies on the leaf extract were carried out against all the juvenile stages of the mosquito, which were grouped based on developmental stage (Table 1). Ten larvae (early or late instar) or pupae were placed in a 150 mL plastic cup with 100 mL of distilled water mixed with 10 mL of leaf extract (Table 1). Five concentrations of leaf extract were administered to the mosquitoes, and each concentration was replicated 15 times. Distilled water was used as a control.

### Efficacy of fruit and leaf powder

Ten larvae/pupae from each group were again placed in a 150 mL plastic cup with 100 mL of distilled water and a given weight (0.1 g, 0.5 g, or 1 g) of fruit or leaf powder (Table 1) was added. Three concentrations of fruit or leaf powder were administered to the mosquitoes and each concentration was replicated 15 times.

Mortalities were recorded every 24 hours until all larvae or pupae had either died or developed to the next stage. To determine mosquito mortality, the surface of the water was disturbed; any larvae or pupae that did not return to the surface after the disturbance were considered dead and were removed. The concentrations of the crude extract in the water containing the juvenile mosquitoes were calculated from the formula: C_1_V_1_ = C_2_V_2_, where C_1_ and C_2_ represent initial and final concentration and V_1_ and V_2_ represent initial and final volume.

**Figure 1.**
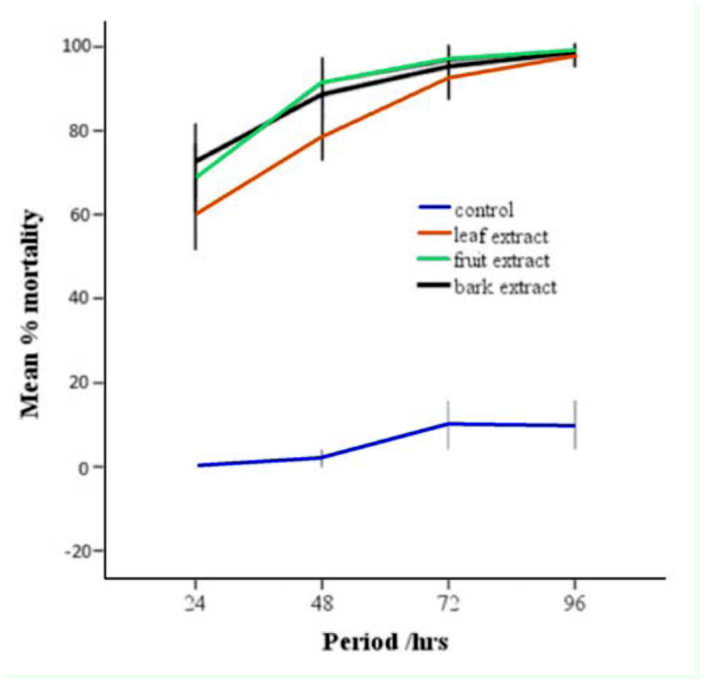
Mean mortality of late larvae of *Culex quinquefasciatus* within a period of exposure to 0.1 g/mL concentration of crude extract from neem leaves, fruits and bark. Error bars represent 2 SE. High quality figures are available online.

**Figure 2.**
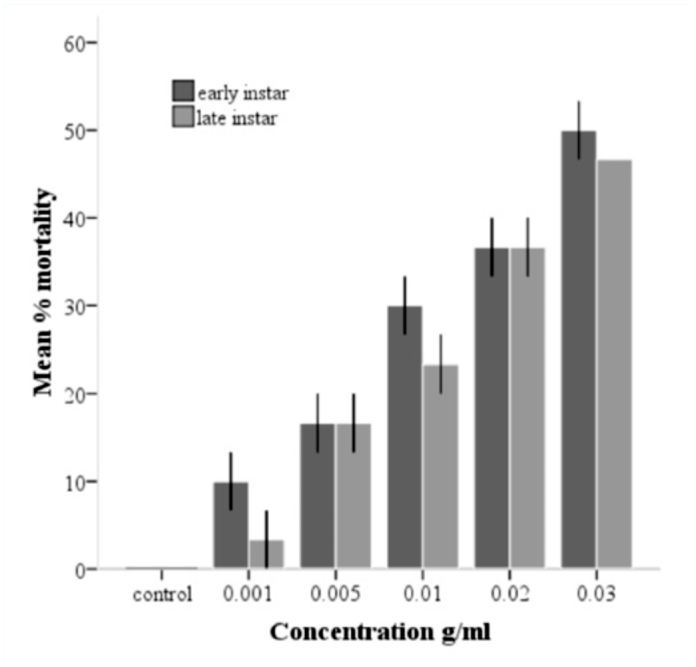
Mean % mortality of early and late instar of *Culex quinquefasciatus* after 24-hour exposure to different concentrations of local extract from neem leaves. Error bars represent I SE. High quality figures are available online.

### Statistical analysis

The data were tested to verify the normality of errors (Shapiro-Wilks test) and homogeneity of variances. Kruskal-Wallis test, as well as multiple comparisons by ranks was used to test the significance of differences between the effects of various concentrations of extract (from different parts of the neem tree) on the mosquitoes. Also, ANOVA was used to analyze the effect of different concentrations of leaf extract on mosquitoes. All statistics were performed by SPSS version 16.

## Results

### Comparative efficacy of fruit, bark, and leaf extract

Aqueous crude extract from the fruit, bark, and leaf caused similar levels of larval mortality (Figure 1). For instance, water containing 0.1 g/mL bark extract caused the highest mean percent larval mortality (± SE) (72.7 ± 1.8) within 24 hours of exposure; this result was not statistically different from the mortality caused by fruit (68.7 ± 1.6) or leaf extract (60 ± 1.6) (χ^2^ = 4.12; df = 2; *p* = 0.127). At higher concentrations, however, bark and fruit extract caused slightly higher mortalities than leaf extract (Tables 2).

**Figure 3A–B.**
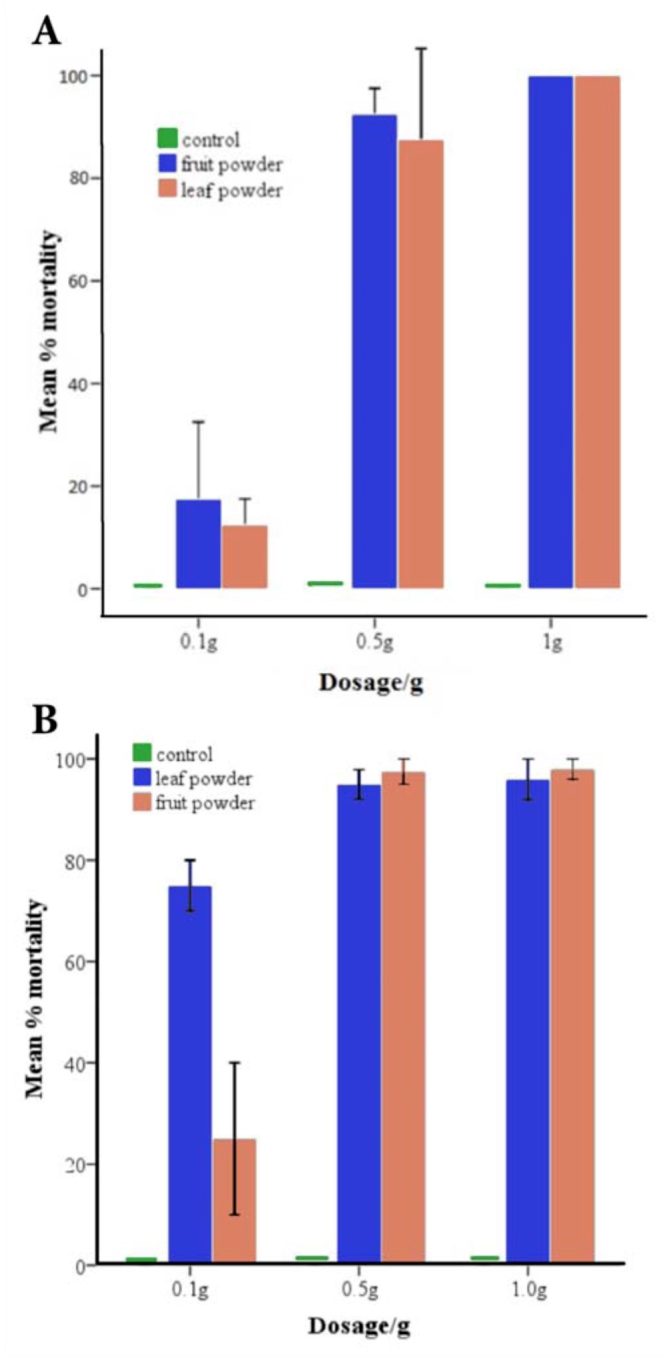
Mean % mortality of (A) late instar larvae and (B) pupae of *Culex quinquefasciatus* after 24-hour exposure to fruit and leaf powder at different doses. Error bars represent I SE. High quality figures are available online.

### Effect of leaf extract on different instars

Early larval instars appeared to be more susceptible to the leaf extract than late larval instars, though this difference in susceptibility was not significant, as indicated by the overlapping standard error bars (Figure 2). When concentration of leaf extract was increased 10×, the period of exposure required to kill > 50% of larvae was reduced by 48 hours (Tables 2 and 3).

Exposure to neem leaf extract either killed pupae of *Cx. quinquefasciatus* or extended the pupal period. In water containing 0.001 g/mL leaf extract, 33.3% of pupae died within 144 hours, while at a higher concentration (0.03 g/mL), 100% of pupae were killed after 120 hours. In the control, 99% of pupae developed to adult within 48 hours.

### Efficacy of fruit and leaf powder

Neem fruit and leaf powder caused similar levels of mortality (Figure 3A). All larvae died within 24 hours of exposure to 1 g of either fruit or leaf powder.

The powders were also potent against pupae; < 5% eclosion occurred after exposure of pupae to 1.0 g of leaf or fruit powder for 48 hours. However, at a dosage of 0.1 g, 25% and 75% emerged as adults within 24 hours of exposure to leaf and fruit powder, respectively (Figure 3B).

### Leaf extract versus leaf powder

**Figure 4.**
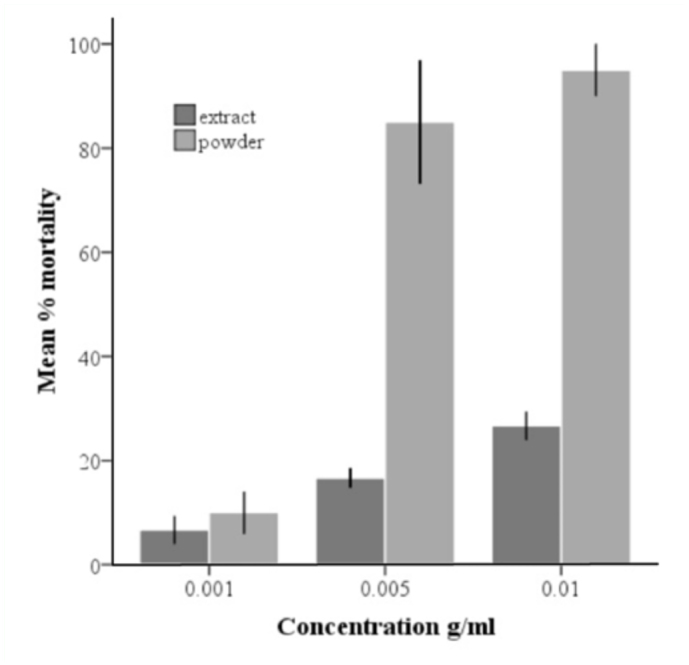
Mean % mortality of *Culex quinquefasciatus* larvae after 24-hour exposure to different concentrations of local extract from neem leaves and neem leaf powder. Error bars represent I SE. High quality figures are available online.

At equal concentration in water, leaf powder caused mortality faster than leaf extract (Figure 4); it took 120 hours for > 95% of larvae to die in leaf extract at 0.01 g/mL, while at equal concentration, leaf powder killed > 95% within 24 hours of exposure. A similar trend was observed when pupae were exposed to powder and extract; at equal concentration (0.005 g/mL), neem leaf powder killed 95% pupae within 24 hours, while in the same time the crude extract killed 13.3%, though pupal mortality in leaf extract increased to 80% after 144 hours.

## Discussion

The results of this study show that larvae and pupae of *Cx. quinquefasciatus* were highly susceptible to crude extract and crude powder made from different parts of the neem tree. Other studies (Howard et al. 2009; Martinez-Tomas et al. 2009) have also demonstrated that crude extracts from different parts of neem are potent against different mosquitoes, including *Cx. quinquefasciatus* and *Anopheles gambiae*. However, this study has shown that crude aqueous extract prepared from bark, fruit, or leaves has a similar effect on *Cx. quinquefasciatus* larvae. Also the effect of powdered neem leaf on larvae and pupae was not different from the effect of powdered fruit. No significant difference was observed between early and late larval instars in their susceptibility to the leaf extract. Howard et al. (2009) made a similar observation on *An. gambiae* when different larval instars were exposed to crude extract from neem bark. In the present study, the leaf extract also had a remarkable effect on the pupae; it prevented the development of pupae to adults or prolonged the pupal period. This extended period of development is still beneficial to mosquito management because it would expose juveniles to predators for longer periods, even if the extract itself did not kill them.

Azadirachtin is one of the main active components of neem and it appears to be responsible for about 90% of the effect on most pests (National Research Council 1992). However, in a related study (Howard et al. 2009), azadirachtin was not present in significant amount in aqueous crude extract from neem bark. In that study, high-performance liquid chromatography analyses of the aqueous crude extract showed the presence of other constituents such as nimbin and salannin. These neem constituents have been found to inhibit ecdysone 20-monooxygenase (an enzyme important for molting) (Mitchell et al. 1997). Therefore mortalities and extended development of the mosquitoes in this study may have been caused by nimbin and salannin. There are several other active compounds in neem (Schmutterer 1990, Mulla and Su 1999) that could also be responsible for the inhibition of growth and development in mosquitoes.

At equal concentrations, neem powder appears to act faster on the mosquitoes than the crude extract. The neem powder floats on water; thus, at higher doses it might have prevented the larvae from breathing, eventually resulting in death by suffocation. Therefore the powder might have acted as a barrier, rather than as a pesticide. In some situations, non-toxic barriers can be effective control agents. For example, when surfaces of pit latrines and cesspits were covered with a 2–3 cm layer of non-toxic expanded polystyrene beads, larvae and pupae were suffocated and female *Cx. quinquefasciatus* were prevented from ovipositing (Curtis et al. 2002).

In Ghana, as in some other parts of Africa, many human activities (especially improper disposal of waste) create anthropogenic breeding sites for mosquitoes. This problem has not been adequately addressed because most of the mosquitoes that breed in anthropogenic habitats (especially *Cx. quinquefasciatus*) are currently not important disease vectors in Ghana. However, their large numbers are becoming an obstacle to the use of insecticide-treated nets and ultimately to malaria control (Kudom and Mensah 2010). Therefore, there is a need to control the populations of these mosquitoes. The results from this study have shown that these anthropogenic breeding sites, mostly in urbanized areas, can be controlled by local preparations of neem as either a crude aqueous extract or a crude powder.

The focus of this study was to find a low-tech control method that could be both used by local people, and integrated into vector control programs. Neem trees are available throughout the country, though fruits are not present year-round, and the continual removal of the bark may be detrimental to the trees. The leaves, however, are always available, easy to harvest, and regenerate quickly. Thus, the use of neem leaves in mosquito control programs appears relatively feasible. Pouring crude extract of leaves in cesspits or other anthropogenic breeding sites may control growth and development of *Cx. quinquefasciatus*. Furthermore, spreading a thick layer of powdered neem leaves in pit latrines can kill *Cx. quinquefasciatus* juveniles and possibly prevent adults from ovipositing. Neem powder might also control flies that breed or feed in pit latrines.

This study explored the use of different local preparations made from different parts of the neem tree on *Cx. quinquefasciatus*. The results show that crude aqueous extract from neem bark, leaves, or fruits have similar effects on the pre-adult stages of this particular mosquito species. The crude extract slowly inhibited growth and development of juveniles, while the crude powder acted more as a barrier, resulting in mortality likely caused by suffocation. However, both types of preparation (powder or leaf extract) can be made and used by local people to control the breeding of mosquitoes in anthropogenic habitats, especially in urbanized areas.

**Table 1.**
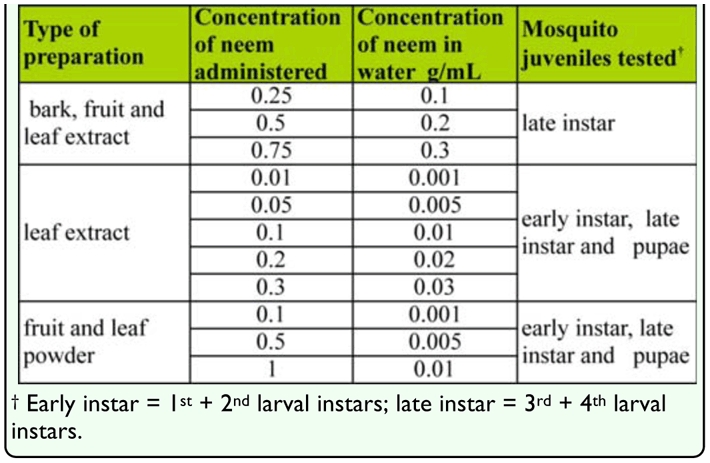
Different concentrations of locally prepared neem g/mL or g used against *Culex quinquefasciatus*.

**Table 2.**
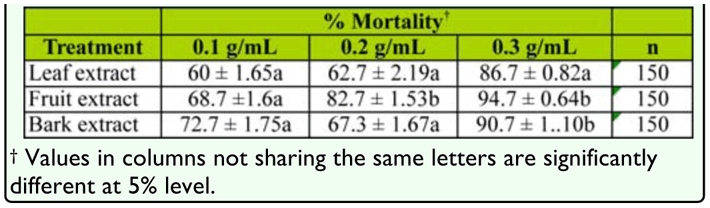
Mean % mortality (± SE) of *Culex quinquefasciatus* larvae after 24-hour exposure to different concentrations of crude extract from neem leaf, fruit and bark.

**Table 3.**
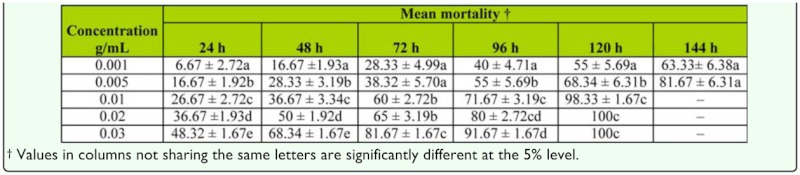
Mean % mortality (± SE) of *Culex quinquefasciatus* larvae after a period of exposure to different concentrations of crude extract from neem leaf.

## References

[bibr01] Awad OM, Shimaila A (2003). Operational use of neem oil as an alternative anopheline larvicide. Part A: laboratory and field efficacy.. *Eastern Mediterranean Health Journal*.

[bibr02] Batra CP, Mittal PK, Adak T, Sharma VP (1998). Efficacy of neem oil-water emulsion against mosquito immatures.. *Indian Journal of Malariology*.

[bibr03] Chandre F, Darriet E, Darder M, Cuany A, Doannio JMC, Pasteur N, Guillet E (1998). Pyrethroid resistance in *Culex quinquefasciatus* from West Africa.. *Medical and Veterinary Entomology*.

[bibr04] Copping LG, Menn J (2000). Biopesticides: a review of their action, applications and efficacy.. *Pest Management Science*.

[bibr05] Corbel V, N'Guessan R, Brengues C, Chandre F, Djogbenou L, Martin T, Akogbeto M, Hougard JM, Rowland M (2007). Multiple insecticide resistance mechanisms in *Anopheles gambiae* and *Culex quinquefasciatus* from Benin, West Africa.. *Acta Tropica*.

[bibr06] Curtis CF, Malecela-Lazaro M, Reuben R, Maxwell CA (2002). Use of floating layers of polystyrene beads to control populations of the filarial vector *Culex quinquefasciatus*.. *Annals of Tropical Medicine and Parasitology*.

[bibr07] Howard AFV, Adongo AEL, Hassanali A, Omlin FX, Wanjoya A, Zhou G, Vulule J (2009). Laboratory Evaluation of the Aqueous Extract of *Azadirachta indica* (Neem) Wood Chippings on *Anopheles gambiae* s.s. (Diptera: Culicidae) Mosquitoes.. *Journal of Medical Entomology*.

[bibr08] Kudom AA, Mensah BA (2010). The potential role of the educational system in addressing the effect of inadequate knowledge of mosquitoes on use of insecticide-treated nets in Ghana.. *Malaria Journal*.

[bibr09] Martínez-Tomás SH, Pérez-Pacheco R, Rodríguez-Hernandez C, Ramirez Valverde G, Ruíz-Vega J (2009). Effects of an aqueous extract of *Azadirachta indica* on the growth of larvae and development of pupae of *Culex quinquefasciatus*.. *African Journal of Biotechnology*.

[bibr10] Mitchell MJ, Smith SL, Johnson S, Morgan ED (1997). Effects of the neem tree compounds azadirachtin, salannin, nimbin and 6-desacetylnimbin on ecdysone 20monooxygenase activity.. *Archives of Insect Biochemistry and Physiology*.

[bibr11] Mulla MS, Su T (1999). Activity and biological effects of neem products against arthropods of medical and veterinary importance.. *Journal of the American Mosquito Control Association*.

[bibr12] National Research Council. (1992). Neem: A tree for Solving Global Problems..

[bibr13] Schmutterer H (1990). Properties and potential of natural pesticides from the neem tree, Azadirachta indica.. *Annual Review of Entomology*.

[bibr14] Service M (2008). *Medical entomology for students*.

[bibr15] Siddiqui BS, Afshan F, Gulzar T, Sultana R, Naqvi SNH, Tariq RM (2003). Tetracyclic triterpenoids from the leaves of *Azadirachta indica* and their insecticidal activities.. *Chemical and Pharmaceutical Bulletin*.

[bibr16] Sukumar K, Perich MJ, Boobar LR (1991). Botanical derivatives in mosquito control: a review.. *Journal American Mosquito Control Association*.

[bibr17] Wandscheer CB, Duque JE, Da Silva MAN, Fukuyama Y, Wohlke JL, Adelmann J, Fontana J. D. (2004). Larvicidal action of ethanolic extracts from fruit endocarps of *Melia azedarach* and *Azadirachta indica* against the dengue mosquito *Aedes aegypti*.. *Toxicon*.

